# The Interim Report of the Fabian Society

**Published:** 1914-03-28

**Authors:** 


### NATIONAL INSURANCE ACT DEVELOPMENTS.

### The Interim Report of the Fabian Society.

With the issue of the *New Statesman* for March 14 a special supplement was presented dealing with the working of the Insurance Act. This supplement consists of the interim report of the Committee of Inquiry instituted by the Fabian Research Department in July last, and it may be said at once that the inquiry is the most comprehensive that has yet been made into the working of the Act. For this reason alone the report demands the closest possible attention, and we shall have to refer to it on many future occasions. But quite apart from the ambitious character of the report, it merits attention by reason of the fact that the Committee of Inquiry may be considered as having been in favour of the general principle of National Health Insurance, while such criticisms as have been made on the administration of the Act are framed with a view to the removal of the causes of dissatisfaction, and the committee has not been reticent in putting forth suggestions and recommendations to give effect to its conclusions.

Before examining the report in detail it should be pointed out that the inquiry was instituted by the Fabian Society with the object of obtaining reliable information not generally available to the public on the administration of the health provisions of the Act, and to enable an impartial examination to be made without political prejudice or bias. Mr. Sidney Webb was the chairman of the committee, which consisted of ninety-five members, all specially chosen on account of the information they were able to obtain. Thus we are told that the committee included eighteen medical men, six actuaries, sixteen friendly society officials, thirteen trade union officials, about twelve barristers, several ex-Civil servants, and a number of members of county, borough, district, or parish councils and of Public Health and Insurance Committees, women as well as men. Their investigations were entirely confined to the administration of the Act in England—they did not extend to Scotland, Ireland, or Wales; but in order that Metropolitan ideas should not preponderate only about half the members of the committee lived in London, the remainder being distributed throughout the provinces. These facts relating to the constitution of the investigating body are given, as it is thought that they lend weight to the conclusions and recommendations.

### "General Success" of National Insurance.

At the outset the committee lay considerable emphasis on the vast proportions of the Insurance scheme, and refer with some indication of amazement to the comparative smoothness with which, they find, the machinery is working. This is illustrated in several ways, which for the moment we must leave unnoticed. They also state quite clearly that they find the general effect of the Act is to relieve personal suffering and to mitigate the dire poverty of numerous families in their hour of need, while it is their belief that the results in connection with public health and infant mortality must be advantageous. In order to make their attitude towards the general scheme quite clear they state: "If in this report attention is given chiefly to points in which the scheme does not appear to be working smoothly, or to the defects and shortcomings which experience is revealing, it is because it is with regard to the detailed alterations and amendments which the National Insurance Acts of 1911 and 1913 require that information and discussion is now most profitable."

The report then proceeds to discuss the very serious allegations that have been made as to the financial soundness of the approved societies, and in this matter, although it is admitted that their information is imperfect, the committee conclude that so far as it goes the information indicates that, contrary to the general expectation, taking the scheme as a whole, men and women together, the aggregate amount paid for sickness and maternity benefits is little, if anything, in excess of what was expected. They go on to point out, however, that this aggregate result fails to convey any idea of the financial situation. There are undoubtedly large numbers of approved societies both for men and women, but especially of those for women, where the claims for sickness benefit have been excessive, and the committee have taken considerable pains to investigate the causes for such excess.

### Actual Excess Sickness.

Malingering and lax administration are each taken in turn as possible causes of the strain on the funds, and while it is admitted that both may have some effect—and possibly in total aggregating a considerable sum—yet the proportion which excess claims arising from such causes bears to the total legitimate claims is so small as to warrant their rejection in the opinion of the committee as the primary reason for excess claims.

The principal cause of the excess claims for sickness benefit over what was anticipated is attributed to an actual excess of sickness. In other words, the committee consider that the sickness rates derived from the experience of the Manchester Unity Friendly Society—though the latest and best experience available—do not furnish an appropriate measure of the rates to be expected in the general mass of the population. In support of their argument they point to the fact that to a very large extent the members of the old friendly societies were picked lives, not only on account of their state of health on joining, but also as regards habits of thrift, regularity of employment, and more than average means. Furthermore, sufficient allowance does not appear to have been made for the feature in the experience of many of the old voluntary societies that certain members did not claim the benefits when they were ill.

The excess claims are particularly evident in the case of women's societies—apart from those inaugurated for a few special classes—and this is attributed principally to incapacity for work arising through pregnancy and maternity.

The contention is thus, by implication, that the excess claims over the actuarial estimates, where they occur, will be a permanent feature of the societies concerned, and it is clearly indicated in the report that the committee consider that it is beyond the power of many of the societies, by any means that they already possess, to remedy the state of affairs. It is all the more important, therefore, that the matter should be faced fairly, and that no effort should be spared to prevent the question being shelved until the insolvency of the societies is so clearly demonstrated that there will be difficulty in rectifying their position. If the committee have done nothing more they have at least shown that it is a matter of urgency that the finances of the societies should be thoroughly investigated, and the necessary steps taken promptly to give effect to the requirements of the situation.

### Administration of Medical Benefit Criticised.

The committee then proceed to make what is, in our opinion, their most trenchant criticism of the administration of the Act—namely, the utter failure of the Government, as represented by the Insurance Commissioners, to carry into effect the provisions of the Act in regard to medical benefit. This criticism of the administration of medical benefit occupies no less than ten foolscap pages, or about one-third of the report; and, in view of the important bearing which the failure of the Commissioners to carry out their statutory duty in this respect is shown to have on the stability of the entire Insurance scheme, the space allotted to the subject appears to be thoroughly warranted.

It is to this section of the report that we desire to draw particular attention.

At the outset the committee state that, on the information before them, no case has been made against the panel practitioners, taken as a whole, though isolated failures of duty, some of which are mentioned, may have occurred, and, indeed, would have been expected to occur in such a vast and hastily improvised service. They point out, however, that the general practitioners who have accepted service on the panels are very unevenly distributed in proportion to population, and that it is in the poorer districts, where the doctors are most needed, that the proportion is least. In spite of the fact that some 70 per cent. of the general practitioners have joined the panels there is a distinct shortage of doctors, and this in itself is held to militate against the Insurance scheme. Moreover, the vaunted "free choice of doctor" has, in the opinion of the committee, aggravated the evil, for, on the whole, no intelligent choice appears to have been exercised, insured persons having added their names to the list, however crowded, of the best-known practitioner in their neighbourhood. Thus, to the uneven distribution of the doctors in proportion to the population is added the uneven allotment of patients to the doctors within the same district, so that in many cases it is a physical impossibility for the panel doctor to give proper attention to his numerous patients.

### Lack of Provision for Serious Illness.

The most serious criticism of the administration of medical benefit is, however, directed to the failure of the Insurance Commissioners to provide for the treatment of serious illness. Medical benefit is defined in the Act as "*adequate* medical attendance and treatment"—attendance at child-birth being alone specifically excluded—and, as pointed out in the most important text-book on the Act by Messrs. Carr, Garnett, and Taylor, "no limitation is placed in the Act on the extent of the treatment," while emphasis is given to the word "adequate" by double reference in the Act itself.

In spite of this fact the Commissioners have contracted with the panel doctors to give treatment "of a kind which can consistently with the best interests of the patient be properly undertaken by a practitioner of ordinary professional competence and skill," and "where the condition of the patient is such as to require services beyond the competence of an ordinary practitioner, the practitioner shall advise the patient as to the steps which should be taken in order to obtain such treatment as his condition may require."

The committee go on to point out "that though the panel doctor is thus to 'advise' the patient what steps to take to get the 'adequate' medical attendance and treatment that the Act promises to him in return for his contributions, *the Commissioners have, except in cases of tuberculosis, taken no steps (and have refused to allow the Insurance Committees to take steps) to provide it.*"

Thus no provision is made for the numerous cases where insured persons require expert treatment, or consultation with a specialist, or institutional treatment. The committee's attitude to the shortage of hospital accommodation will be dealt with next week.

